# Left atrial mechanical remodelling assessed as the velocity of left atrium appendage wall motion during atrial fibrillation is associated with maintenance of sinus rhythm after electrical cardioversion in patients with persistent atrial fibrillation

**DOI:** 10.1371/journal.pone.0228239

**Published:** 2020-01-29

**Authors:** Paweł Wałek, Janusz Sielski, Iwona Gorczyca, Joanna Roskal-Wałek, Katarzyna Starzyk, Elżbieta Jaskulska-Niedziela, Radosław Bartkowiak, Beata Wożakowska-Kapłon

**Affiliations:** 1 Intensive Cardiac Care Unit, Świętokrzyskie Cardiology Center, Kielce, Poland; 2 Faculty of Medicine and Health Sciences, The Jan Kochanowski University, Kielce, Poland; 3 Świętokrzyskie Cardiology Center, Department of Cardiology and Electrotherapy, Kielce, Poland; The University of Hong Kong, HONG KONG

## Abstract

The velocity of left atrium appendage (LAA) wall motion during atrial fibrillation (AF) is a potential marker of mechanical remodelling. In this study, we investigated whether the velocity of LAA wall motion during AF predicted the success of electrical cardioversion and long-term sinus rhythm maintenance. Standard echocardiographic data were obtained by transthoracic echocardiography, and LAA wall motion velocities were measured by transoesophageal echocardiography. With logistic regression and receiver operating characteristic curve analyses, we related echocardiographic and clinical data to cardioversion outcomes and sinus rhythm maintenance at 12 months. Of 121 patients prospectively included in the study, electrical cardioversion restored sinus rhythm in 97 (81.2%), and 51 (42%) patients maintained sinus rhythm at 12 months. Patients in whom cardioversion restored sinus rhythm had higher LAA wall motion velocities than did the patients with failed cardioversions (p <0.001). Compared to patients with AF at 12 months, patients who maintained sinus rhythm had lower maximum and end-diastolic left atrial volumes (p ≤ 0.01), lower E/e’ ratios (p = 0.005), higher s’ values (p = 0.013), and higher LAA motion velocities (p < 0.001). On multivariate logistic regression, only LAA wall motion velocity and E/e’ ratios remained significant predictors of sinus rhythm maintenance at 12 months (p ≤ 0.04). LAA wall motion velocity was also a significant predictor of sinus rhythm maintenance when corrected for clinical variables (p = 0.039). Conclusion: LAA wall motion velocity, as a marker of mechanical remodelling, can predict short-term and long-term sinus rhythm maintenance after electrical cardioversion in AF.

## Introduction

Atrial fibrillation (AF) is the most common supraventricular arrhythmia.[1] Depending on its symptoms, AF is managed with restoration of sinus rhythm (rhythm control) or maintenance of heart rate (rate control).[2] Sinus rhythm can be effectively restored by pharmacological or electrical cardioversion. In the Euro Heart Survey, the effectiveness of cardioversion was 75%-88%, and the percentage of patients who maintained sinus rhythm over 12 months was 70%.[3]

Changes in atrial structure or function, also referred to as atrial remodelling, may increase the risk of recurrence or persistence of AF. There are three main types of atrial tissue remodelling: electrical, structural, and mechanical. Atrial remodelling has an important place in the pathophysiology of supraventricular arrhythmias, including AF, and other heart diseases such as mitral valve defects, heart failure, ischemic heart disease, and channelopathies. Thus, many investigators are looking for imaging and laboratory markers of atrial remodelling that could help assess the clinical progression of atrial cardiomyopathies.[4]

Different groups have reported on potential clinical, imaging, and laboratory predictors of sinus rhythm maintenance after cardioversion of AF.[5–8] To assess the mechanical function of the left atrium (LA) or left atrial appendage (LAA) by echocardiography, we can use the assessment of the left atrial emptying fraction (LA EF), measurement of blood flow velocity through the mitral valve, strain or strian rate using tissue doppler technique imagine (TDI) or speckle tracking echocardiography (STE) and measurements of left atrial appendage emptying velocity (LAAEV).[9–23] Currently, much attention is paid to the use of STE technique to assess the mechanical function of LA and LAA, however, due to the limitations of this method, i.e. obtaining optimal visualization, especially in the middle and distal segments of LAA, we decided to use the TDI technique. Previous studies using TDI showed that non-invasive measurements of atrial motion during AF were reliable markers of mechanical remodelling that predicted successful cardioversions.[24–26] Recently, Farese et al. showed that LAA wall velocity assessment using TDI can help identify patients with paroxysmal AF.[23] However, there is scarce evidence regarding the importance of LAA wall motion velocity during AF for long-term cardioversion outcomes. Therefore, we investigated whether LAA wall motion velocity was associated with maintenance of sinus rhythm 12 months after successful electrical cardioversion of AF.

## Methods

### Study population

The study protocol was approved by the ethics committee of Świętokrzyska Medical Chamber. This prospective observational study enrolled 121 patients with persistent AF who underwent direct current cardioversion between August 2015 and May 2017 on a cardiology ward. The inclusion criteria were as follows: symptomatic persistent atrial fibrillation for ≥ 7 days, ejection fraction >40%, and effective anticoagulation with warfarin, acenocumarol, or novel oral anticoagulants (dabigatran, rivaroxaban, apixaban) for ≥3 weeks before cardioversion. The exclusion criteria were as follows: age < 18 years, no consent to participate in the study, no consent for cardioversion or transesophageal echocardiography, low quality of echocardiographic images, moderate or severe valve regurgitation or stenosis, valvular prosthesis, presence of thrombus in the LAA, acute decompensation of heart failure, acute myocardial infarction, previous pulmonary vein isolation, dysthyroidism, anaemia (haemoglobin < 6.9 mmol/l), and cancer.

Clinical and electrocardiographic follow-up was obtained for all patients with sinus rhythm at months 1, 6, and 12. A 24-h ambulatory electrocardiographic monitoring was performed in all patients who had sinus rhythm at 1 and 12 months. Patients were asked to report to our Department when they felt palpitations or thought that AF had recurred.

### Clinical data

Clinical data were obtained on the day of cardioversion and included age, sex, body mass index (BMI), body surface area (BSA, calculated with the Gehan and George formula), glomerular filtration rate (GFR, calculated with the Cockroft-Gault formula), hypertension, diabetes mellitus, dyslipidemia, smoking status, history of coronary artery disease, European Heart Rhythm Association (EHRA) score, dysthyroidism, obstructive pulmonary disease, renal disease, and history of stroke or transient ischemic attack. Coronary artery disease was diagnosed when patients had a history of myocardial infarction, percutaneous coronary intervention, or coronary artery by-pass grafting. Data regarding AF duration and the duration of the current AF episode were recorded only when patients could accurately determine these data. Because patients often did not know when the episode of AF started, these data were available for only 84 patients (69.4%). CHA2DS2-VASc and HAS-BLED scores were recorded according to the current European guidelines on AF treatment.[2]

### Restoration of sinus rhythm

All cardioversions were performed with anaesthesiological assistance under general sedation. All cardioversions were performed with a biphasic defibrillator (150–300 J). When the first shock was ineffective, the next attempt was performed with a higher energy (by 100 J). The success of cardioversion was defined as sinus rhythm maintenance for ≥ 24 hours. Patients with sinus rhythm received anticoagulants, up-stream therapy, or antiarrhythmic drugs by clinical judgment. Antiarrhythmic drugs, like amiodarone and propafenone, were prescribed by a physician blinded to echocardiography results.

### Transthoracic echocardiography

Transthoracic echocardiography was performed with the Vivid S6 Echocardiographic system (General Electric Medical Systems, Horten, Norway), equipped with the M4S RS transducer. One experienced echocardiographer performed all examinations according to current guidelines.[27, 28] Standard M-mode images, Doppler images, and 2-dimentional cine loops in the parasternal long and short axes and apical 2-, 3-, and 4-chamber views were obtained in every patient. All images and measurements were acquired from standard views and stored digitally. The stored echocardiographic images were retrieved and analysed with off-line software (EchoPAC PC software, GE Medical Systems). The maximum end-systolic volume of the left atrium (LAV) and the minimum end-diastolic volume of the left atrium (LAEDV) were measured by the Simpson’s method from apical 4- and 2-chambers views. The maximum LAV was measured at the end of systole, on the frame just before mitral valve opening, by tracing the inner border of the atrium and taking care to avoid the area under the valve annulus, appendage, and pulmonary veins. LAV was indexed to BSA (LAVI). The minimum LAEDV was measured at the end of ventricular diastole, on the frame of mitral valve closure, and indexed to BSA (LAEDVI). Left ventricular (LV) volume and ejection fraction (LVEF) were calculated with the Simpson’s formula. The area of the right atrium was measured in the apical 4-chamber view at the end of systole (RAAs, right atrium area at systole) and at the end of diastole (RAAd, right atrium area at diastole), on the frame with tricuspid valve closure. Blood flow velocities were measured by transmitral pulsed wave Doppler (PWD) from the apical 4-chamber view with a 2-mm sample volume placed between the tips of the mitral leaflets. Tissue Doppler imaging (TDI) of the mitral annulus motion was performed from the apical 4-chamber view with a 5-mm sample volume at the lateral and septal basal regions. S’ mean and e’ mean values were calculated as averages from septal and lateral measurements. The measurements taken during atrial fibrillation were calculated by averaging data from 5 consecutive beats.

### Transesophageal echocardiography

**Fig 1:**
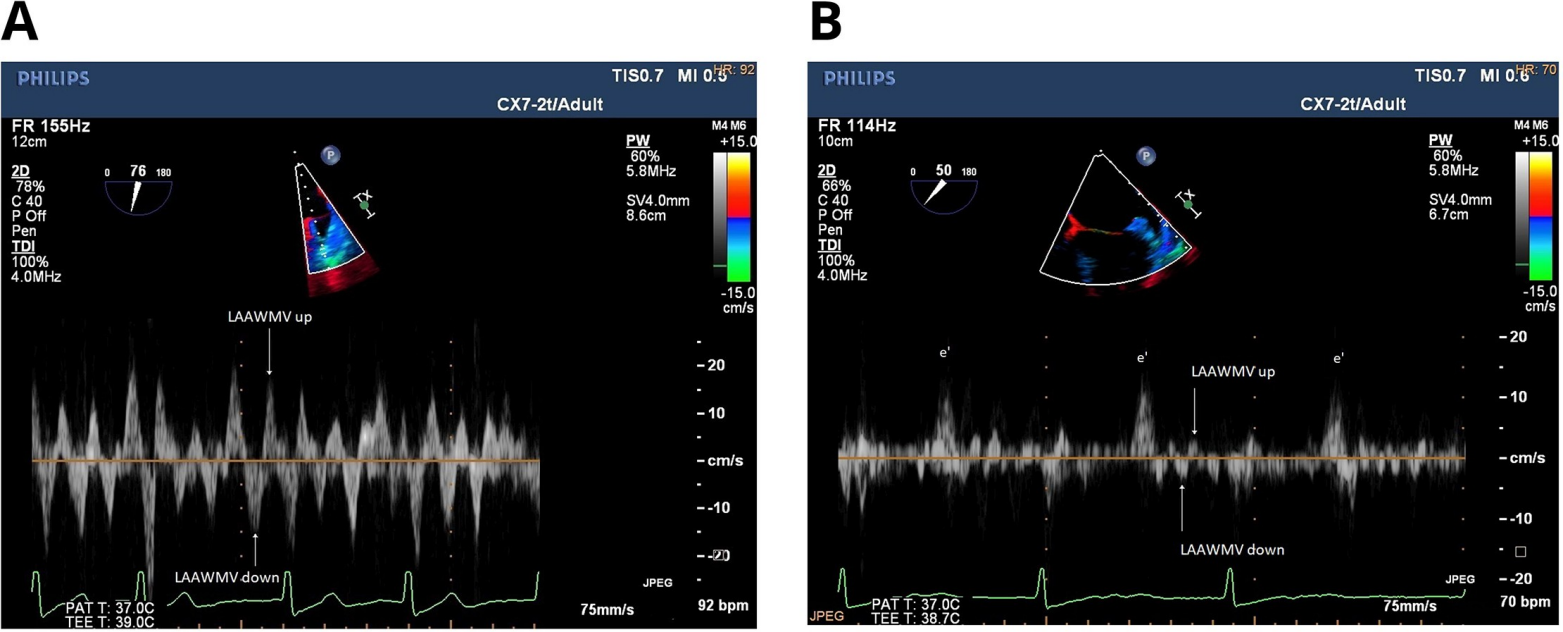
Example measurements of left atrial appendage wall motion velocity: high velocities (a) and low velocities (b). On the Fig 1B, the wave corresponding to the e' wave of diastolic motion of the mitral annulus is visible.

Transesophageal echocardiography was performed on the day of or one day before cardioversion with the Phillips iE33 echocardiograph (Phillips Medical Systems, Andover, MA) equipped with a 7-MHz transesophageal model X7-2t. The study was conducted in accordance with the current recommendations of the European Association of Echocardiography.[29] LAA was evaluated for thrombi, noting spontaneous echo contrast. LAA was imaged in the lower and upper transoesophageal views with a multiplane 2D technique and adjustment of gain settings. Measurements of LAAEV were taken in LAA about 2 mm from the ostium. Next, the measurements of the LAA wall motion velocity (LAAWMV) were performed in the two-chamber 60–90° projection with tissue Doppler and a sample volume of 4mm; velocity measurements were taken for about 3 seconds. The view angle was obtained by positioning the analysis segment of LAA wall as much as possible along the direction of the ultrasound wave propagation. Medial and lateral LAA wall apart from moving towards and away from the echocardiography probe move also centripetally to the LAA cavity. The LAA tip moves mainly towards and away from the echocardiography probe because the centripetal move to the LAA cavity is simultaneously a move towards the echocardiography probe. LAAWMV was measured at the apex of LAA (LAAWMV apex), mid-length of the lateral wall (LAAWMV lateral), and mid-length of the medial wall (LAAWMV medial). LAAWMV was measured based on AF waves recorded just before QRS complexes, after the wave corresponding to the e' wave of the diastolic motion of the mitral annulus. The measurements of the highest LAAWMV towards the probe (LAAWMV up) and from the probe (LAAWMV down) and the highest velocity to or from the probe (LAAWMV max) were analysed (Fig 1A and 1B).

### Statistical analysis

Results are described as means ± standard deviations (SD). Categorical variables are presented as counts and percentages. Normally distributed variables were compared with the Student’s t-test, and non-normally distributed variables were compared with the Mann-Whitney test or the chi-squared test.

Receiver-operated characteristic (ROC) curves for predicting sinus rhythm maintenance at 1, 6, and 12 months were calculated for selected echocardiographic variables. Optimal cutoffs were calculated based on the Youden’s statistic, and areas under the curve (AUC) were compared with the DeLong test with the AUC that indicated no diagnostic values (0.5). From the LAAWMV evaluated, a parameter with the best AUC for twelve-month observation was selected for the multivariable stepwise and forward analysis (LAAWMV apex MAX with AUC 0.738). We analysed predictors of sinus rhythm maintenance with univariate logistic regression. Significant echocardiographic predictors from univariate analysis (p < 0.1) were analysed with multivariate stepwise and forward logistic regression to avoid multicollinearity. The stepwise inclusion was set at p < 0.05 and exclusion at p>0.1. Moreover, we analysed predictors of sinus rhythm maintenance with a logistic regression model that included age, sex, hypertension, GFR, use of specific medications, and LAAWMV at apex, which had the greatest AUC for predicting sinus rhythm maintenance from echocardiographic parameters.

Thirty patients were randomly identified for intraobserver and interobserver agreement. Intraobserver and interobserver reproducibility were computed by median absolute percentage error and intraclass correlation coefficient (ICC) with 95% confidence intervals. Significance was set at p < 0.05. Statistical analyses were performed with MedCalc Statistical Software version 18.6 (MedCalc Software bvba, Ostend, Belgium).

## Results

**Fig 2:**
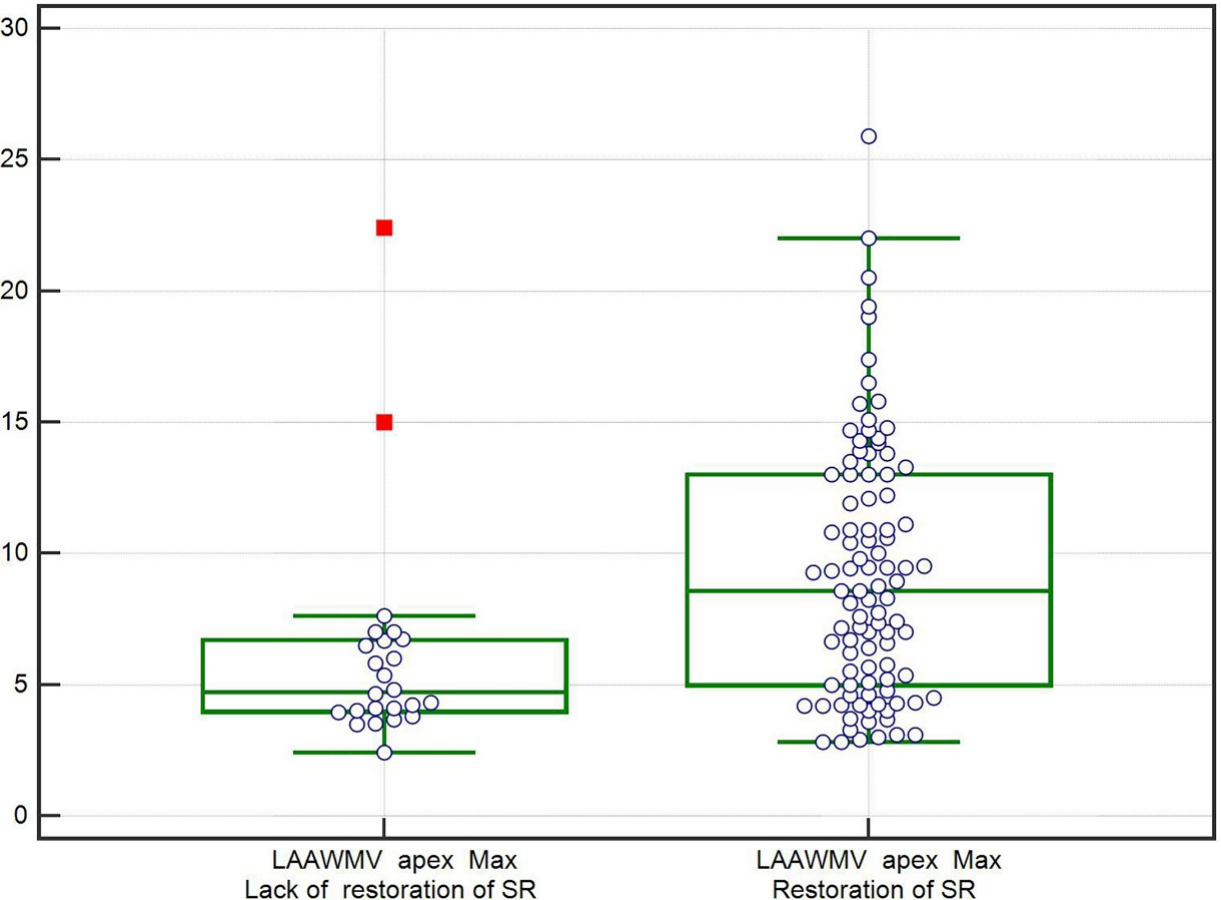
Boxplot presenting maximum LAAWMV values at apex in patients with or without restoration of sinus rhythm.

Of 121 patients included in the study, electrical cardioversion restored sinus rhythm in 97 (81.2%). At 12 months, 51 (42%) patients maintained sinus rhythm. Compared with patients who had unsuccessful cardioversions or AF recurrence, patients who maintained sinus rhythm over 12 months had higher GFR values, used beta-blockers more often before cardioversion, used spironolactone/eplerenone more often before and after cardioversion, and used diuretics less often before and after cardioversion (p ≤ 0.044 for all comparisons, Table 1). There were no other significant differences in baseline characteristics between patients who maintained sinus rhythm over 12 months and the remaining patients (Table 1). Patients with successful cardioversions had significantly higher values of LAAWMV at apex (mean± SD, 9.2±4.8 cm/s), than those with unsuccessful cardioversions (6.1±4.9 cm/s; p < 0.001, Fig 2).

**Table 1:** Baseline characteristic of sinus rhythm maintenance after 12 months. Clinical parameter characteristics: Pre–drugs taken before cardioversion. Post–drugs taken after cardioversion.

	Study population n 121	SR maintenance n 51 (42.1%)	Failure of DCCV or recurrence of AF n 70 (57.9%)	p
Age (years)	64.3±10.3	62.4±12	65.7±8.6	0.231
Age <65, n (%)	53 (43.8)	26 (51)	27 (38.6)	0.176
Age 65–74, n (%)	54 (44.6)	19 (37.3)	35 (50)	0.166
Age ≥75, n (%)	15 (12.4)	7 (13.7)	8 (11.4)	0.706
Males, n (%)	77 (63.6)	37 (72.5)	40 (57.1)	0.083
BMI (kg/m2)	30.2±4.8	30.3±4.1	30±5.3	0.369
Total AF duration (months)*	10.6±17.8	9.5±20.9	11.2±16.1	0.161
AF duration current episode (weeks)*	13.2±20.4	12.3±11.4	13.6±24	0.104
Number of previous electrical cardioversions (n)	0.4±0.8	0.3±0.7	0.4±0.8	0.564
Hypertension, n (%)	99 (81.8)	40 (78.4)	59 (84.3)	0.412
Diabetes mellitus, n (%)	25 (20.7)	10 (19.6)	15 (21.4)	0.808
Coronary artery disease, n (%)	19 (15.7)	8 (15.7)	11 (15.7)	0.997
EHRA scale III-IV, n (%)	39 (32.2)	17 (33.3)	22 (31.4)	0.826
EHRA, mean	2.5±0.8	2.5±08	2.5±0.8	0.982
Stroke/TIA, n (%)	13 (10.7)	5 (9.8)	8 (11.4)	0.777
Vascular disease, n (%)	14 (11.6)	7 (13.7)	7 (10)	0.529
CHA2DS2-VASC, mean	2.7±1.6	2.5±1.6	2.8±1.7	0.227
CHA2DS-VASC = 0, n (%)	8 (6.6)	3 (5.9)	5 (7.1)	0.784
CHA2DS2-VASC = 1, n (%)	24 (19.8)	13 (25.5)	11 (15.7)	0.185
CHA2DS-VASC ≥2, n (%)	89 (73.6)	35 (68.6)	54 (77.1)	0.296
HAS-BLED scale, mean	0.4±0.7	0.3±0.5	0.5±0.8	0.067
Smokers, n (%)	12 (9.9)	4 (7.8)	8 (11.4)	0.516
GFR (ml/min)	85.2±28.5	93±31	78.9±24.9	0.021
Beta-blockers pre, n (%)	107 (88.4)	50 (98)	57 (81.4)	0.005
Amiodarone pre, n (%)	10 (8.3)	2 (3.9)	8 (11.4)	0.140
ACE inhibitors/ARB pre, n (%)	100 (82.6)	42 (82.4)	58 (82.9)	0.943
Spironolactone/eplerenone pre, n (%)	23 (19)	14 (27.5)	9 (12.9)	0.044
Statins pre, n (%)	79 (65.3)	36 (70.6)	43 (61.4)	0.298
Diuretics pre, n (%)	57 (47.1)	16 (31.4)	41 (58.6)	0.003
Amiodarone post, n (%)	39 (32.2)	15 (29.4)	24 (34.3)	0.573
Beta-blockers post, n (%)	99 (81.8)	45 (88.2)	54 (77.1)	0.120
Propafenone post, n (%)	30 (24.8)	15 (29.4)	15 (21.4)	0.317
ACE inhibitors/ARB post, n (%)	100 (82.6)	42 (82.4)	58 (82.9)	0.943
Statins post, n (%)	78 (64.5)	32 (62.7)	46 (65.7)	0.737
Diuretics post, n (%)	60 (49.6)	16 (31.4)	44 (62.9)	0.001
Spironolactone/eplerenone post, n (%)	25 (20.7)	15 (29.4)	10 (14.3)	0.043

AF, atrial fibrillation; BMI, body mass index; GFR, glomerular filtration rate; TIA, transient ischemic attack; ACE inhibitors/ARB, Angiotensin-converting-enzyme inhibitors/Angiotensin II receptor blockers; Pre, before cardioversion; Post, after cardioversion

*data available for 88 patients only.

Compared with patients who had AF recurrence, patients who maintained sinus rhythm over 12 months had lower values of LAVI, LAEDV index, E wave and E/e’ ratios (p < 0.05 for all comparisons), and they had higher values of e’ and s’ waves, LAAEV and LAAWMV in all locations examined (p < 0.001 for all comparisons, Table 2). There were no other significant differences in echocardiographic variables between patients who maintained sinus rhythm over 12 months and the remaining patients (Table 2). On univariate logistic regression, significant echocardiographic predictors of sinus rhythm maintenance over 12 months included LAVI, LAEDV index, s’ wave values, e’ wave values, E wave values, E/e’ ratios, LAAWMV at apex, and LAAEV (Table 3). On multivariate stepwise logistic regression, E/e’ ratios and LAAWMV at apex remained significant independent predictors of sinus rhythm maintenance at 12 months (Table 3).

**Table 2:** Characteristics of echocardiographic parameters obtained before cardioversion.

	Study population n 121	SR maintenance n 51 (42.1%)	Failure of DCCV or recurrence of AF n 70 (57.9%)	p
RV prox (mm)	30.8±3.9	31.6±4.2	30.2±3.6	0.053
IVS (mm)	10.8±1.8	10.7±1.8	10.9±1.8	0.767
LVEDD (mm)	51.5±6.6	51.9±6.7	51.2±6.6	0.552
LVESD (mm)	36±7.6	36.7±8.1	35.5±7.5	0.381
LVEDV (ml)	120.9±35.8	127.6±36.9	115.9±34.3	0.076
LVESV (ml)	54.2±21.6	56.9±20.7	52.2±22.2	0.237
LVSV (ml)	66.1±20.6	68.7±20.9	64.2±20.4	0.202
LVEF (%)	56±10.7	54.6±9.9	57.1±11.2	0.291
RVSP (mmHg)	29.5±11.5	28.8±9.9	30±12.7	0.685
LA AP (mm)	44.2±4.5	43.5±3.8	44.7±4.9	0.139
LAVI (ml/m^2^)	47.9±13	44.4±12.1	50.5±13.1	0.010
LAEDV Index (ml/m^2^)	35.1±12.7	31.1±11.5	38±12.9	0.001
RAA d (cm^2^)	22.2±4.9	22.4±5.2	22.7±4.1	0.164
RAA s (cm^2^)	16.6±4.2	16.1±4.3	16.9±4.2	0.139
s’ mean (cm/s)	6.1±1.7	6.5±1.8	5.7±1.6	0.013
e’ mean (cm/s)	9.9±2.4	10.6±2.3	9.4±2.4	0.011
E/e’ mean	9.7±4.2	8.4±2.8	10.8±4.8	0.006
E (m/s)	0.9±0.2	0.8±0.2	0.9±0.2	0.006
LAAWMV apex up max (cm/s)	6.5±3.4	7.5±2.9	5.8±3.6	0.0001
LAAWMV apex down max (cm/s)	8.4±4.9	10.1±4.1	7.2±5.1	< 0.0001
LAAWMV apex max (cm/s)	8.6±4.9	10.3±4.1	7.3±5	< 0.0001
LAAWMV lateral up max (cm/s)	5.8±2.7	7±3.1	5±2	0.0003
LAAWMV lateral down max (cm/s)	7.2±4.2	8.6±4.2	6.2±4	0.0003
LAAWMV lateral max (cm/s)	7.4±4.1	8.9±4.1	6.4±3.9	0.0003
LAAWMV medial up max (cm/s)	5.4±3.1	6.5±3.4	4.6±2.6	0.0003
LAAWMV medial down max (cm/s)	7.5±4.8	8.9±5.6	6.4±3.9	0.0004
LAAWMV medial max (cm/s)	7.6±4.8	9.2±5.5	6.5±3.8	0.0001
LAAEV max (cm/s)	39.4±17.8	47.1±19.7	33.8±14	0.0001

E, early filling wave; e’, early diastolic mitral annular velocity; LA AP, left atrial antero-posterior; LAAWMV, left atrial appendage wall motion velocity; LAAEV, left atrial appendage emptying velocity; LAEDVI, left atrial end-diastolic volume index; LAVI, left atrial volume index; LVEDD, left ventricular end-diastolic diameter; LVESD, left ventricular end-systolic diameter; LVEDV, left ventricular end-diastolic volume; LVEF, left ventricular ejection fraction; LVESV, left ventricular end-systolic volume; LVSV, left ventricular stroke volume; IVS, intraventricular septum wall thickness; RAA, right atrium area, d–diastolic, s–systolic; RV prox, right ventricular proximal diameter; RVSP, right ventricular systolic pressure; s’, systolic mitral annular velocity

**Table 3:** Echocardiographic determinants of sinus rhythm maintenance for 12 months. Stepwise and forward multivariable regression analysis. Echocardiographic parameters obtained before cardioversion with P<0.1 in univariate regression analysis. Prognosis of maintenance of SR for 12 months.

	Univariate analysis			Multivariable analysis		
	OR	95% CI	p-value	OR	95% CI	p-value
LAVI (ml/m^2^)	0.961	0.930–0.992	0.014			
LAEDV Index (ml/m^2^)	0.952	0.920–0.985	0.005			
s’ mean (cm/s)	1.335	1.057–1.687	0.015			
e’ mean (cm/s)	1.241	1.046–1.474	0.014			
E/e’ mean	0.841	0.748–0.944	0.004	0.877	0.776–0.991	0.036
E (m/s)	0.082	0.011–0.637	0.017			
LAAWMV apex max (cm/s)	1.144	1.051–1.244	0.002	1.096	1.004–1.196	0.040
LAAEV (cm/s)	1.027	1.007–1.048	0.007			

CI, confidence interval; E, early filling wave; e’, early diastolic mitral annular velocity; LAAEV, left atrial appendage emptying velocity; LAAWMV, left atrial appendage wall motion velocity; LAEDVI, left atrial end-diastolic volume index; LAVI, left atrial volume index; OR, odds ratio; s’, systolic mitral annular velocity.

On univariate logistic regression, clinical predictors of sinus rhythm maintenance over 12 months included GFR, beta-blockers use before cardioversion, and diuretic and spironolactone/eplerenone use after cardioversion (p ≤ 0.046, Table 4). In a multivariate logistic regression model including clinical variables and LAAWMV, only beta-blockers use before cardioversion, diuretic use after cardioversion, and higher LAAWMV values remained significant predictors of sinus rhythm maintenance at 12 months (p ≤ 0.039, Table 4).

**Table 4:** Echocardiographic and clinical determinants of sinus rhythm maintenance for 12 months. Multivariable regression analysis.

	Univariate analysis			Multivariable analysis		
	OR	95% CI	p-value	OR	95% CI	p-value
Age (years)	0.968	0.933–1.004	0.080			
Males	1.982	0.912–4.307	0.084			
Hypertension	0.678	0.268–1.713	0.411			
GFR (ml/min)	1.032	1.008–1.057	0.009			
Amiodarone post	0.799	0.367–1.740	0.571			
Beta-blockers pre,	11.404	1.440–90.293	0.021	23.556	2.308–240.411	0.012
Propafenone post,	1.528	0.666–3.503	0.317			
Diuretics post,	0.270	0.126–0.580	0.001	0.340	0.131–0.880	0.026
Spironolactone/eplerenone post,	2.500	1.016–6.152	0.046			
LAAWMV apex max (cm/s)	1.144	1.051–1.244	0.002	1.121	1.006–1.249	0.039

CI, confidence interval; GFR, glomerular filtration rate; LAAWMV, left atrial appendage wall motion velocity; OR, odds ratio; post, after cardioversion; pre, before cardioversion.

**Fig 3:**
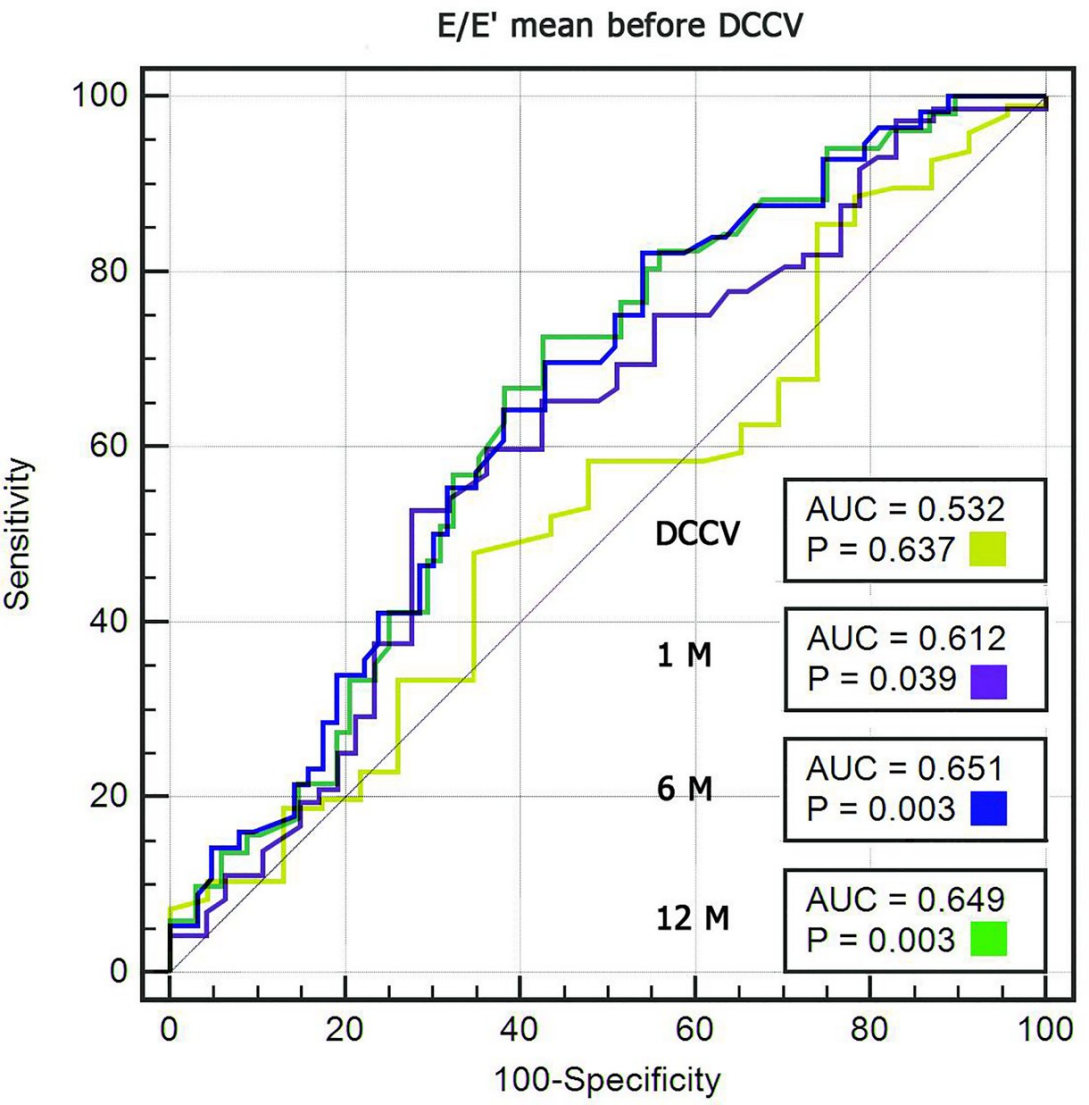
Prediction of successful cardioversion and sinus rhythm maintenance at 1, 6, and 12 months. Receiver operating characteristic curves for the E/e’ ratio measured before cardioversion. AUC, (area under the curve); p values for comparisons with no diagnostic vale (AUC = 0.5, DeLong test).

On ROC curve analysis, E/e’ ratios were not a significant predictor of sinus rhythm restoration by cardioversion (AUC = 0.532, p = 0.637), but they were a significant predictor of sinus rhythm maintenance over 12 months (AUC = 0.649, p = 0.003, Fig 3). Based on the Youden’s statistic, the optimal E/e’ cutoff for predicting sinus rhythm maintenance was 8.72, with sensitivity of 72.55% and specificity of 57.35%.

**Fig 4:**
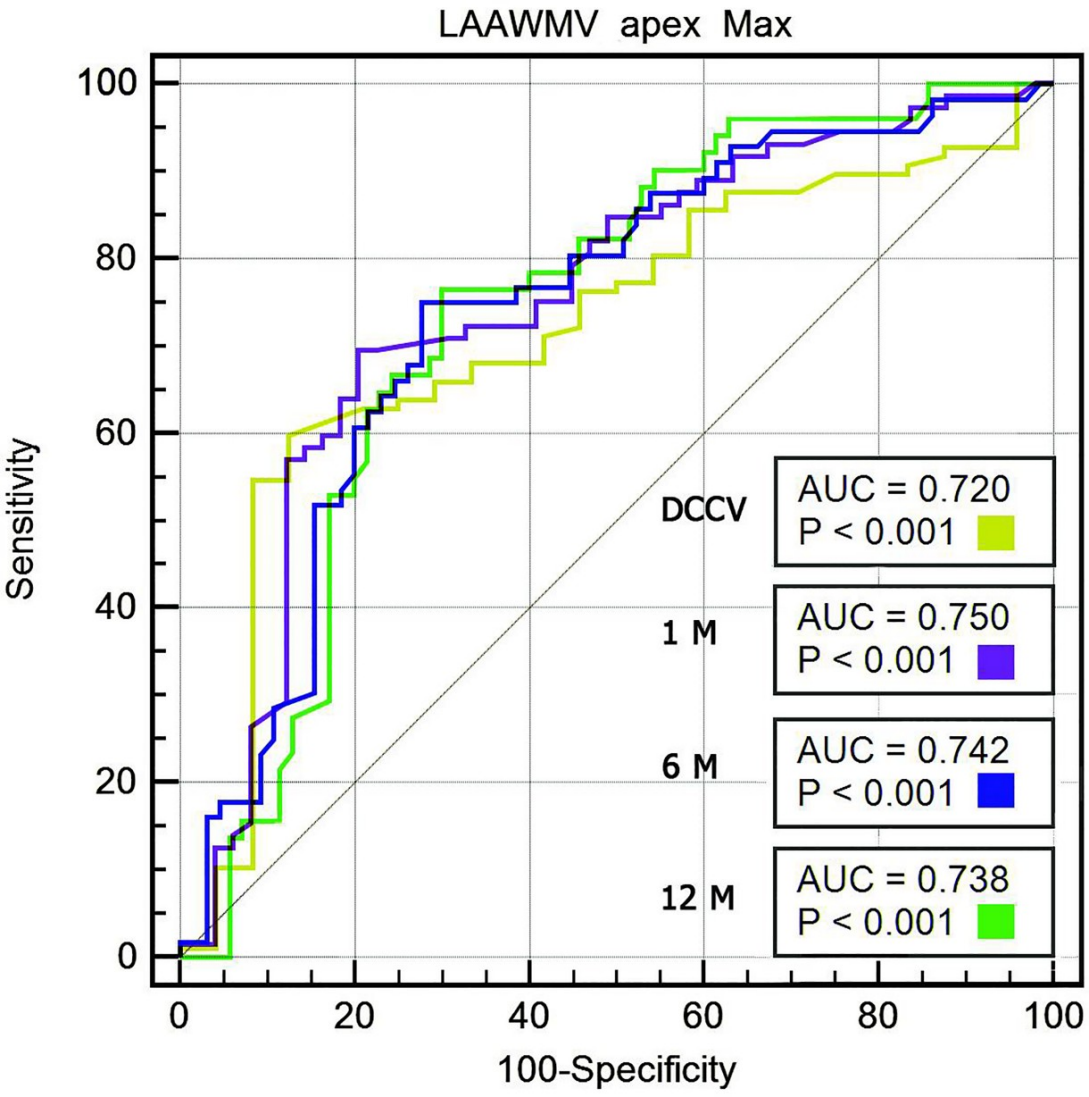
Prediction of successful cardioversion and sinus rhythm maintenance at 1, 6, and 12 months. Receiver operating characteristic curves for maximum LAAWMV at apex. p values for comparisons with no diagnostic value (AUC = 0.5, DeLong test).

On ROC curve analysis, LAAWMV at apex was a significant predictor of both restoration of sinus rhythm (AUC = 0.720, p < 0.001) and sinus rhythm maintenance over 12 months (AUC = 0.738, p < 0.001, Fig 4). Based on the Youden’s statistic, the optimal cutoff of LAAWMV at apex was 7.16 cm/s, with sensitivity of 76.5% and specificity of 70%. In a multivariate logistic regression model including echocardiographic parameters from Table 3 odds ratio for sinus rhythm maintenance with LAAWMV apex max >7.16 cm/s was 7.8 (95% CI 3.34–17.908; p<0,001). Multivariate logistic regression analysis with LAAWMV apex max >7.16 cm/s corrected for clinical variables from Table 4 OR for sinus rhythm maintenance was 6.318 (95% CI 2.555–15.622; p<0.001).

### Reproducibility

Intraobserver correlation coefficients and median absolute percentage error for LAAWMV apex max were 0,996 (95% CI 0,992–0,998) and 3,65% (95% CI 2,79–4,58), respectively. Interobserver correlation coefficients and median absolute percentage error for the same parameter were 0,995 (95% CI 0,985–0,998), respectively.

## Discussion

In this study, we found that LAA wall motion was a significant predictor of successful electrical cardioversion and long-term maintenance of sinus rhythm in patients with AF. Thus, our findings suggest that LAA wall motion is a clinically important marker of atrial fibrosis.

Atrial fibrosis is an important substrate of mechanical remodelling because it decreases contractility, leads to atrial enlargement, and increases the recurrence risk of AF.[30] In this study, we investigated whether LAA wall motion was a clinically valid marker of mechanical remodelling. One of the advantages of LAA wall motion over other markers of mechanical remodelling, like left atrial ejection fraction, is that it can be measured before cardioversion. In this study, we found that patients with sinus rhythm maintenance and those with recurrence of atrial fibrillation differed with respect to echocardiographic markers of structural remodelling (LAVI, LAESV Index), mechanical remodelling (LAAWMV apex, LAAEV), LV filling pressure (E/e’ ratio), and wave s’ values. However, on multivariate analyses, only LAAWMV and E/e’ ratios remained significant predictors of sinus rhythm maintenance at 12 months. Unlike E/e’ ratios, LAAWMV was also a significant predictor of successful cardioversion. Thus, our study suggests that mechanical remodelling is more important than structural remodelling for predicting cardioversion outcomes. Our results are in line with previous reports.

Di Salvo et al., using TDI, found that patients with a greater deformation of the left atrial wall were more likely to maintain sinus rhythm after electrical cardioversion of recent-onset lone atrial fibrillation.[9] In another study, patients with failure of cardioversion or recurrence of AF within 4 weeks had lower deformation of basal LA wall compared to both patients who maintained sinus rhythm and patients without AF.[10] The superiority of the STE strain measurement over the STE motion measurement is based mainly on its independence on the angle of the ultrasound beam. The major limitation of the STE strain measurement is its dependence on the imaging quality of the given structure which in case of thin and difficult to visualize LAA wall is of high importance. We think that the assessment of the LAA wall strain measurement can be done in LAA ostial segments. Application of the LAA wall strain analysis using STE needs further research especially in the context of a diagnosis of patients with atrial fibrillation.

De Vos et al. found that higher atrial fibrillatory wall velocities were associated with short-term and long-term success of electrical cardioversion.[25] Moreover, patients with a longer AF duration tended to have lower atrial fibrillatory velocities, which suggested that LAA wall motion velocity reflected mechanical remodelling.[11, 26] In line with previous research, we found that higher LAA wall velocities were associated with successful cardioversions and sinus rhythm maintenance in patients with AF. Uretsky et al. showed that LAA wall contraction velocity measured by TDI was decreased in patients with LAA thrombus, AF, spontaneous echo contrast, and with previous stroke or transient ischemic attack.[14] Recently, Farese et al. showed that patients whose LAA side wall velocity is lower than the median wall are patients with paroxysmal AF or at risk of AF.[23]

LAAWMV can also be used to monitor changes in remodeling. For example, LAA wall contractility improved after percutaneous mechanical mitral commissurotomy or ablation of persistent AF.[16,17]

Similar to LAAWMW, atrial fibrillatory contraction flow could be related to mechanical activity of the left atrium during AF and be a marker of mechanical remodelling. Kim et al. reported that the presence of left atrial fibrillatory contraction flow, measured immediately after early diastolic mitral inflow during AF, predicted long-term success of electrical cardioversion.[12] In a study among 3251 patients with first-time successful transoesophageal-guided cardioversion, LAAEV was independently associated with maintenance of sinus rhythm, ischaemic stroke, and mortality over 12 years.[13] Luong et al., who assessed mechanical atrial remodelling during sinus rhythm, showed that right and left atrial emptying fraction after successful electrical cardioversion were better predictors of sinus rhythm maintenance than were right and left atrial volume indices.[15] These investigators also showed that markers of mechanical remodelling were better predictors of sinus rhythm maintenance than the markers of structural remodelling. Our findings confirm this observation.

Our study had limitations. It was carried out in one centre and included a small sample. Moreover, the echocardiographic techniques we used require substantial skill from the operator. Measuring LAAWMV requires a pause in the ventricular systolic function to visualize the LAA wall contraction wave; however, this pause may be shorter than suggested by De Vos et al. based on transthoracic echocardiography.[24, 25] When patients have a very fast ventricular function, it is difficult to measure LAAWMV. However, transoesophageal echocardiography, via vagal nerve stimulation, often slows heart rate and enables reliable LAAWMV measurements. We did not re-evaluate LAAWMV after successful DCCV or after 12 months of follow-up; therefore, we do know whether this method can be used to monitor changes in LAA remodeling. We assessed AF duration retrospectively based on patients’ reports. Because this method is unreliable, we did not analyse AF duration as a predictor of sinus rhythm maintenance after successful cardioversion. Moreover, because using constant heart rhythm monitoring was not feasible in our long-term study, we could have missed self-limiting episodes of AF recurrence. No LA or LAA deformation analysis was performed, and in the light of new reports this could provide additional information regarding fibrosis and mechanical function of LA and LAA. We performed all cardioversions in the antero-lateral position, without changing paddle position in case of cardioversion failure, which might have influenced the success rate.

In conclusion, our findings support the view that markers of mechanical remodelling of the left atrium are important for predicting sinus rhythm maintenance after electrical cardioversion in patients with AF. In our study, higher LAAWMVs increased the likelihood of sinus rhythm maintenance independently of concomitant diseases. We showed that markers of mechanical remodelling are better predictors of sinus rhythm maintenance than the markers of structural remodelling or LV filling. Assessing atrial remodelling can help predict outcomes of electrical cardioversion and enable a better qualification of patients with AF for rhythm control therapy.
